# TRIM59 promotes steatosis and ferroptosis in non-alcoholic fatty liver disease via enhancing GPX4 ubiquitination

**DOI:** 10.1007/s13577-022-00820-3

**Published:** 2022-11-22

**Authors:** Jingxian Zhang, Haina Xie, Jun Yao, Wenye Jin, Huijie Pan, Zhiqiang Pan, Dongyu Xie, Donghao Xie

**Affiliations:** 1 Department of Pharmacy, Shanghai Guanghua Hospital of Integrative Medicine, Shanghai, China; 2 School of Basic Medical Sciences, Shanghai University of Traditional Chinese Medicine, Shanghai, China; 3 Department of Spleen-Stomach, Zhenjiang Affiliated Hospital of Nanjing University of Chinese Medicine, Zhenjiang, China; 4 Department of Spleen-Stomach, Zhenjiang Hospital of Traditional Chinese Medicine, Zhenjiang, China; 5 Institute of Arthritis Research in Integrative Medicine, Shanghai Academy of Traditional Chinese Medicine, Shanghai, China

**Keywords:** Non-alcoholic fatty liver disease, Ferroptosis, TRIM59, GPX4, Ubiquitination

## Abstract

Non-alcoholic fatty liver disease (NAFLD) is the most common liver disease around the world. However, no specific medicine has been approved for NAFLD treatment. Our study was conducted to explore the role and mechanism of TRIM59 in NAFLD, aiming to provide a novel target for NAFLD treatment. Here, the expression of TRIM family members was detected in 10 mild and severe NAFLD tissues as well as 10 normal tissues. TRIM59 expression was verified in 10 normal tissues and 25 mild and severe NAFLD tissues. Palmitic acid and high-fatty diet were used for the construction of NAFLD models. Oil Red O staining was used to detect the level of steatosis. The content of TNF-α, IL-6, and IL-8 was measured to reflect the level of inflammation. Lipid reactive oxygen species was estimated by flow cytometry. We found that TRIM59 was highly expressed in NAFLD tissues compared with normal liver tissues. The inhibition of TRIM59 could inhibit the steatosis and inflammation in NAFLD, whereas its overexpression exhibited reversed effects. The application of ferroptosis inhibitor, deferoxamine, could markedly ameliorate steatosis and inflammation, which was mediated by overexpressed TRIM59. Besides, TRIM59 was demonstrated to interact with GPX4 and promoted its ubiquitination. The overexpression of GPX4 could significantly reverse the pathogenic effects of TRIM59 in NAFLD. Additionally, the inhibition of TRIM59 appeared to be a promising strategy to ameliorate NAFLD in mice model. In summary, our study revealed that TRIM59 could promote steatosis and ferroptosis in NAFLD via enhancing GPX4 ubiquitination. TRIM59 could be a potential target for NAFLD treatment.

**Supplementary Information:**

The online version contains supplementary material available at 10.1007/s13577-022-00820-3.

## Introduction

Non-alcoholic fatty liver disease (NAFLD) is a lifestyle-related chronic liver malady and the number of cases around the world is increased in recent times. Based on severity and acuteness of disease and symptoms, it could be classified as initial steatosis to steatohepatitis to cirrhosis or advanced fibrosis. Among these, non-alcoholic steatohepatitis (NASH) is the most severe NAFLD. In non-alcoholic patients, NASH symptoms include incidence of lobular inflammation, lipid accumulation in hepatic tissues, and degenerative ballooning [1–3]. Only few clinical studies are available, and there is lack of information regarding the details of molecular switch and progression of NAFLD to NASH. Recent findings show several factors could cause the disease [2, 4] including genetic and environmental factors. Moreover, dysfunction of gut, liver and adipocyte-derived adipokines is also predicted to play important role in the development of NASH. Preliminary data suggest that steatosis and lipo-toxicity could be caused by the availability of saturated fatty acids, including palmitic acid (PA), leading to NASH [5–7]. Additionally, saturated free fatty acid activates toll-like receptor (TLR) responses resulting in macrophage inflammation [8]. It is widely known that lipo-toxicity is responsible for insulin resistance, hepatocyte dysfunction, fibrogenesis, and inflammation. Hence, the atonement of lipo-toxicity and oxidative stress could be a convincing strategy to overcome the incidence of NASH [9].

The TRIM (tripartite motif-containing) family comprises over 70 member proteins, containing a RBCC motif (RING domain, B-box domains and a coiled-coil domain) [10]. Members of the TRIM family actively participate in several processes, such as transcription, cellular protection, cell division, and differentiation [11]. Various studies show members of the TRIM family, including TRIM25, TRIM24, TRIM19 and TRIM13 are involved in liver cancer, leukemia, breast, and gastric and hepatic inflammation [12–15]. Moreover, expression of TRIM24 was found to suppress development of spontaneous hepatic fatty acid accumulation and hepatocellular carcinoma [13, 15, 16]. Whereas, TRIM16 is found to play a preventive role in the development of neuroblastoma and cell migration [17]. A novel member of TRIM family, TRIM59 is engaged in specific human cancers, like liver cancer and hepatic disorders [18, 19]. Furthermore, TRIM59 is also implicated in liver inflammation and carcinogenesis by enhancing the ubiquitination and degradation of p53 [20] and TRIM59 may stimulate lung cancer cells without interfering activity of p53 [21].

NASH is an advanced stage of NAFLD having higher fatty acid accumulation in liver cells and causes hepatic cell death, inflammation and fibrosis too [22]. Moreover, NASH is discovered to be among the rapidly emerging hepatic disorders [23]. It is reported that ferroptosis, a relatively novel disorder, is believed to trigger cell death by an iron-dependent and oxidative stress leading to NASH. Glutathione peroxidase (GPX) proteins are one of the key regulatory proteins with peroxidase activity and having major biochemical function in protection of membrane and tissues from oxidative damage. The GPX4 pathway is implicated in steatosis and ferroptosis, GPX4 is a selenoprotein and it was the first identified as a central inhibitor of ferroptosis [24]. GPX4 decreases the availability of lipid peroxides [25] via glutathione (GSH) and stimulates ferroptosis [26]. Few studies found that non-functional GPX4 (either deleted or inactive form of GPX4) is embryonically lethal [27, 28]. On the contrary, Ingold et al. reported the selenolate-based GPX4 is not necessary for normal embryogenesis [29]. In knockout mice models, the lipid peroxidation was enhanced and may cause embryonic death. Additionally, Yang et al. found either overexpression or knockdown of GPX4 can control cell death ferroptosis inducers but cannot protect from other mechanisms [24]. Several findings implicate the role of GPX4 in the regulation of ferroptosis [24, 29, 30]. Hence, GPX4 may be looked upon as a significant therapeutic agent for organ deteriorating disorders (upregulated GPX4) and tumorigenic disorders (loss of GPX4).

The specific role of TRIM59 in steatosis or ferroptosis as well as regulation of GPX4 is not known. In current study, we observed highly expression of TRIM59 in NAFLD tissues, and then elucidated the promoting role of TRIM59 in cell lipid or fat accumulation with palmitic acid (PA)-treated L02 cells. We also found that TRIM59 plays key role in the ubiquitination of GPX4, which further leads to ferroptosis. Additionally, we further evaluated these findings in vivo based on a high-fatty diet-induced NAFLD mouse model.

## Materials and methods

### Data extraction

Gene expression of NAFLD tissues was extracted from GEO database (GSE49541 [31], http://www.ncbi.nlm.nih.gov). Differentially expressed genes (DEGs) in the mild and severe groups were identified by GEO2R (Table 1).

**Table 1:** Gene expression analysis of TRIM family members by GEO2R with GSE GSE49541

Gene	Log FC	Ave Expr	*t*	*P* value	Adj *P* value
TRIM59	0.293533976	1.949238	4.332629	4.60E-05	0.001926
TRIM69	0.296526594	6.388445	2.92084	0.004635	0.057051
TRIM9	0.324151569	2.249059	2.664059	0.009485	0.089181
TRIM6	0.324781113	3.512708	3.124103	0.002553	0.037998
TRIM22	0.415040682	8.302295	2.931385	0.004496	0.056053

*FC* fold change

### NAFLD clinical specimens

This study was approved by the Institutional Ethics Review Board (Shanghai University of Traditional Chinese Medicine, Shanghai, China; Approval number: 2022-K-50) and conducted according to the ethical guidelines of the 1975 Declaration of Helsinki. Our study included 50 patients with NAFLD and 12 control subjects. Exclusion criteria were extensive alcohol use (> 20 g/day), positive testing for hepatitis B virus surface antigen (AgHBs) and hepatitis C virus (HCV) antibodies. The NAFLD patients were divided into mild (fibrosis stage 0 or 1, *n* = 25) and severe (fibrosis stage 3 or 4, *n* = 25) based on fibrosis staging system as previously described [32]. Liver biopsies and serum samples were collected for further analysis. Written informed consent was obtained from patients.

### Enzyme-linked immunosorbent assay (ELISA) analysis

The content of ferritin, transferrin (TRF), TNF-α, IL-6 and IL-8 in serum or culture medium was determined with ELISA kits (Jiancheng Biothech., Nanjing, China).

### Measurement of triglycerides (TG), Fe^2+^, aspartate aminotransferase (AST) and alanine aminotransferase (ALT)

The concentrations of TG, Fe^2+^, AST and ALT in serum samples were measured with kits from Jiancheng Biothech (Nanjing, China) according to the manufacturer’s protocols.

### Cell cultures

Human liver cell line (L02) and murine liver cell line (AML12) were obtained from Shanghai Biology Institute. Cells were maintained in RPMI-1640 medium with 10% fetal bovine serum (Invitrogen) at 37 °C with 5% CO_2_.

### RNA isolation and quantitative real-time PCR

Total RNA of cells and liver tissues was extracted using Trizol reagent (Invitrogen). Gene expression was determined using SYBR^®^Green (Thermo Fisher Scientific) on ABI 7300 (Applied Biosystems). The expression of β-actin was taken as the control. The expression of gene was calculated by 2^−△△CT^ method. Data were represented with the mean of three independent replicates. All primers used in this study are listed in Table S1.

### Western blot

Total protein was extracted using RIPA buffer containing proteinase inhibitor (Beyotime). Proteins were separated in 10% SDS-PAGE and transferred onto nitrocellulose membranes (Millipore, Billerica, WI, USA). After the application of primary and secondary antibodies, the enhanced chemiluminescence system (ECL) was used to detect the protein content. All primary antibodies are listed in Table S2.

### Construction of TRIM59 adenovirus and lentivirus

Short hairpin RNA (shRNA) was designed targeting human (Table S3) and mouse (Table S4) TRIM59 with control shRNA (shNC). The plasmid was recombined with backbone pAdEasy-1 to construct pAd-shTRIM59. Then they were transfected into 293 cells to produce adenovirus. The full-length human TRIM59 and GPX4 were cloned into pLVX-Puro vector (Clontech, Palo Alto, CA, USA). The lentivirus was produced in 293T cells along with packaging plasmids.

### Oil red O (ORO) staining

Cells were cultured on coverslips in 24-well plates. Cells were fixed with 4% formaldehyde for 10 min and stained with ORO solution (Nanjing Jiancheng Bioengineering Institute, Nanjing, China) for 15 min. The nuclear was stained with hematoxylin.

### Lipid peroxidation assay using flow cytometry

The level of lipid reactive oxygen species (ROS) was determined by C11-BODIPY assay. Cells were treated with 10 mM C11-BODIPY-containing medium for 1 h. Then flow cytometry (FACSCantoTM II, BD Biosciences) was used to detect the lipid ROS level.

### Co-immunoprecipitation (Co-IP) assays

Cell lysates were reacted with anti-TRIM59 (Novus Biologicals, NBP1-59777), anti-GPX4 (Proteintech, 14432–1-AP) or control IgG (Santa Cruz Biotech., Santa Cruz, CA, USA) for 1 h at 4 °C. Precipitates were collected using protein A/G-agarose and detected by western blot.

### Animal research

Animal protocols were approved by the Hospital Institutional and Local Animal Care and Use Committee (Approval number: LS21-Feb-R025), and all the animal experiments were performed following the committee’s guidelines. A mouse NAFLD model was constructed by high-fat diet (HFD) feeding method. Eighteen C57BL/6 mice were randomly divided into three groups: group I, normal diet + shNC; group II, HFD + shNC; group III, HFD + shTRIM59 (*n* = 6 in each group). NAFLD was induced in groups II and III, and then shNC and shTRIM59 provirus (1 × 10^8^ pfu per mouse) were injected tail vein into mice in groups II and III, respectively, every 5 days. Mice in group I were fed with normal diet and shNC adenovirus was injected tail vein. After 8 weeks of treatment, all mice were sacrificed and the blood was collected for subsequent ELISA and biochemical analysis. Livers were immediately excised for histological examination and immunoblotting. For histological examination, liver tissues were stained with hematoxylin and eosin (HE) and Oil Red O. All procedures were performed following our animal care guidelines.

### Statistical analysis

All the in vitro experiments were repeated three times independently. Statistical analysis was conducted using Graphpad Prism version 6.0 (CA, USA). Student’s t and one-way ANOVA were used to estimate the difference between two groups or more than two groups, respectively. *P* value < 0.05 was statistically significant.

## Results

### TRIM59 was highly expressed in NAFLD tissues

**Fig. 1:**
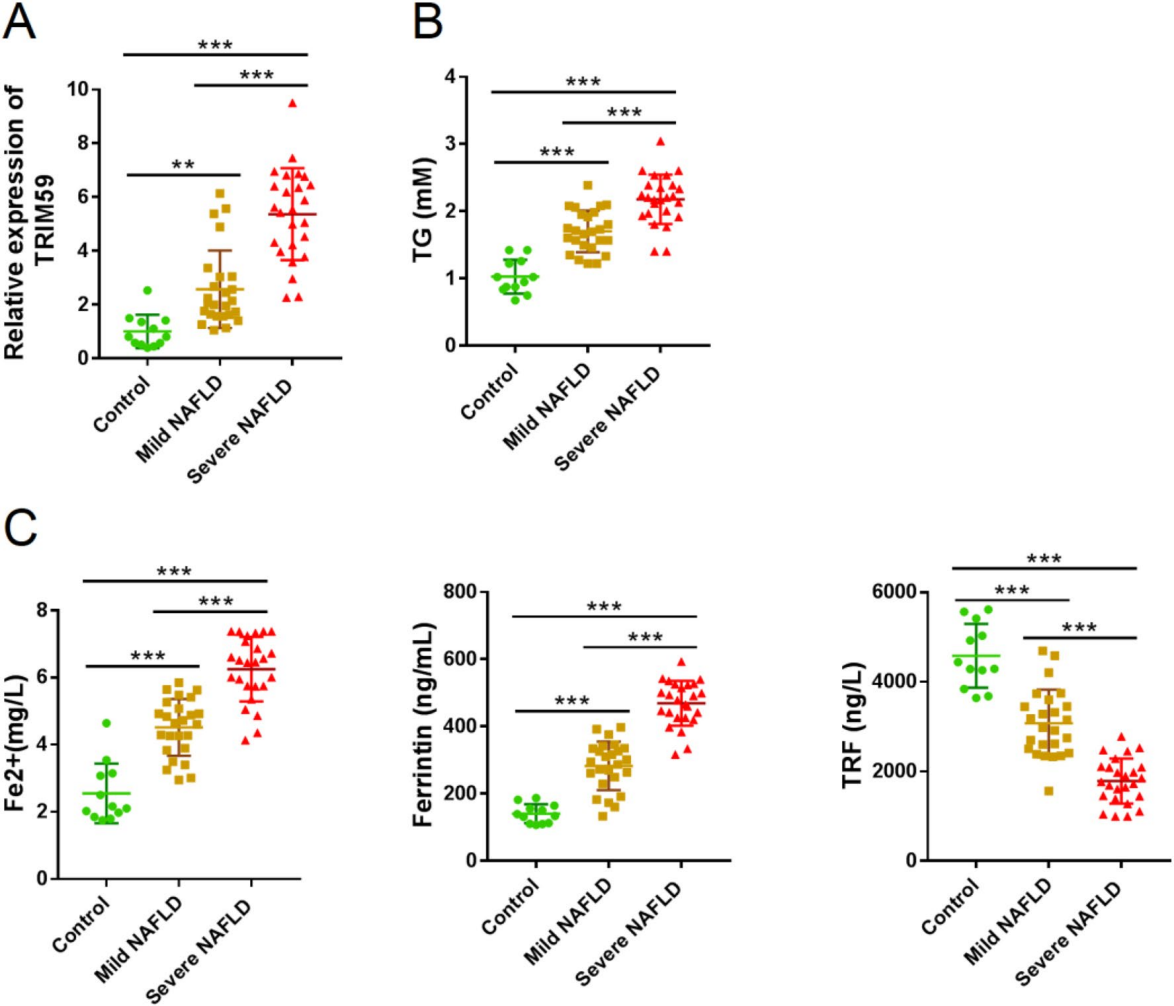
TRIM59 was highly expressed in NAFLD tissues. A total of 12 normal liver tissues, 25 mild fatty liver tissues, and 25 severe fatty liver tissues were collected. **A**–**C** The mRNA level of TRIM59 (**A**) in collected tissues, and serum levels of TG (**B**), Fe^2+^, ferritin, and TRF (**C**). **P < 0.01, ***P < 0.001. *TG* triglycerides, *TRF* transferrin

To explore the role of TRIM family in NAFLD, we analyzed DEGs in a public available dataset, GSE49541 [31]. We found that TRIM6, TRIM9, TRIM22, TRIM59, and TRIM69 was highly expressed in severe NAFLD tissues as compared to mild NAFLD tissues (Table 1). Then the expression of these 5 TRIM genes was detected in 10 normal liver tissues, 10 mild NAFLD tissues and 10 severe NAFLD tissues. The expression of TRIM59 was significantly elevated in NAFLD tissues compared with normal liver tissues, whereas its expression was highest in severe NAFLD tissues (*P* < 0.05) (Figure S1). Further we collected 12 normal liver tissues, 25 mild NAFLD tissues, and 25 severe NAFLD tissues by biopsy and confirmed the change trends of TRIM59 mRNA (Fig. 1A). Moreover, the expression of TG, Fe^2+^, and ferritin was significantly elevated in NAFLD tissues than normal tissues, with the highest level in severe NAFLD tissues (*P* < 0.05) (Fig. 1B, C); meanwhile, the expression of TRF was significantly decreased in NAFLD tissue and even reduced in severe NAFLD tissues (*P* < 0.05) (Fig. 1C). These results indicated that TRIM59 and ferroptosis might contribute to the development of NAFLD.

### Knockdown of TRIM59 inhibited PA-induced steatosis and inflammation

**Fig. 2:**
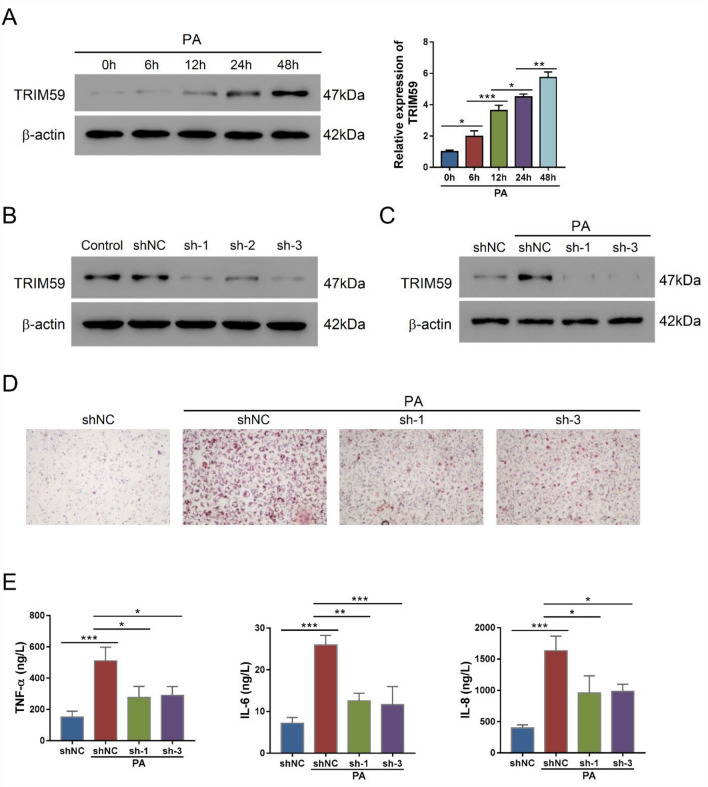
Knockdown of TRIM59 inhibited PA-induced steatosis and inflammation in L02 cells. **A** The expression of TRIM59 in L02 cells treated with 0.4 mM palmitic acid (PA) for 0–48 h. **B** The expression of TRIM59 in L02 cells transduced with three shTRIM59 adenovirus. **C** The expression of TRIM59 in L02 cells transduced with shTRIM59 and treated with 0.4 mM PA. **D** Oil red O staining detected the level of steatosis in L02 cells treated with shTRIM59 and 0.4 mM PA (magnification: 200×). **E** The level of TNF-α, IL-6, and IL-8 in L02 cells treated with shTRIM59 and 0.4 mM PA. *P < 0.05, **P < 0.01, ***P < 0.001, *n* = 3

PA is a steatogenic agent and routinely applied to evoke steatosis in cultured hepatic cells [33]. Herein, we applied PA in L02 cells to mimic NAFLD, in which TRIM59 expression was found to significantly elevate in a time-dependent manner (*P* < 0.05) (Fig. 2A). To explore the potential role of TRIM59 in NAFLD cell model, we adopted three adenovirus (shTRIM59) to suppress the expression of TRIM59, which markedly inhibited the expression of TRIM59 in L02 cells with the presence of PA or not (Fig. 2B, C). ORO staining revealed that the application of PA notably promoted the level of steatosis, whereas the knockdown of TRIM59 could reverse this effect (Fig. 2D). Meanwhile, PA would promote the secretion of inflammatory cytokines, such as TNF-α, IL-6 and IL-8, whereas the knockdown of TRIM59 could significantly decrease their secretion (*P* < 0.05) (Fig. 2E). These findings suggested that the inhibition of TRIM59 could suppress steatosis and inflammation in NAFLD cell model.

### Overexpression of TRIM59 exacerbated PA-induced steatosis and inflammation

**Fig. 3:**
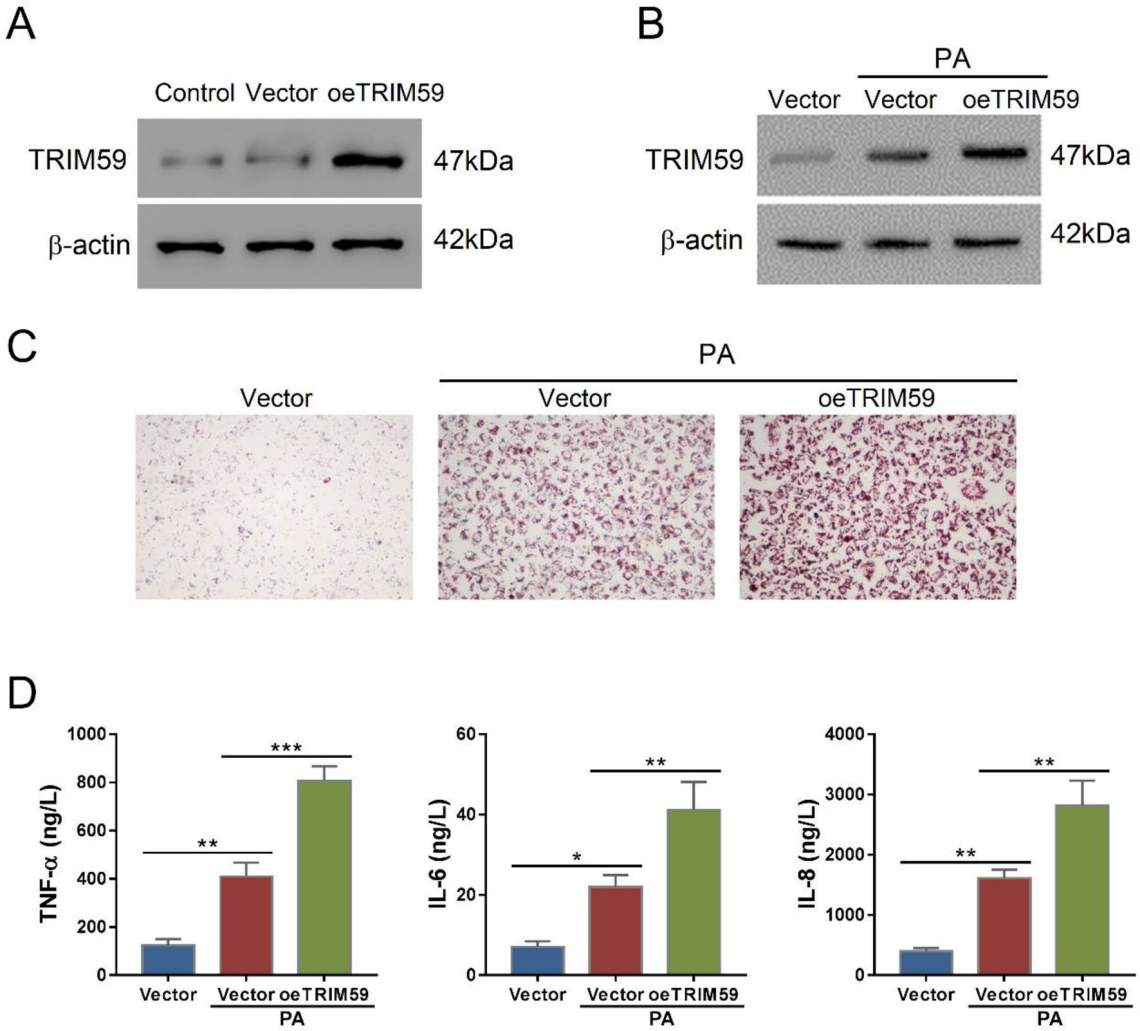
TRIM59 overexpression exacerbates PA-induced steatosis and inflammation in L02 cells. **A**, **B** The expression of TRIM59 in L02 cells transduced with overexpressed TRIM59 lentivirus (oeTRIM59) with the presence of palmitic acid (PA) or not. **C** Oil red O staining detected the level of steatosis in L02 cells treated with oeTRIM59and 0.4 mM PA (magnification: 200×). **E** The level of TNF-α, IL-6, and IL-8 in L02 cells treated with oeTRIM59 and 0.4 mM PA. *P < 0.05, **P < 0.01, ***P < 0.001, *n* = 3

To further verify the role of TRIM59 in NAFLD, we transduced TRIM59 overexpressed lentivirus (oeTRIM59) in L02 cells, which markedly promoted the expression of TRIM59 (Fig. 3A). The application of PA increased the expression of TRIM59 and oeTRIM59 further promoted its expression (Fig. 3B). In L02 cells, PA notably increased the level of steatosis whereas overexpressed TRIM59 further promoted the steatosis (Fig. 3C). Meanwhile, PA significantly increased the secretion of TNF-α, IL-6 and IL-8, whereas the overexpression of TRIM59 further promoted their secretion (*P* < 0.05) (Fig. 3D). These results indicated that TRIM59 could promote PA-induced steatosis and inflammation in NAFLD cell model.

### Deferoxamine inhibited TRIM59-induced steatosis and inflammation in L02 cells

**Fig. 4:**
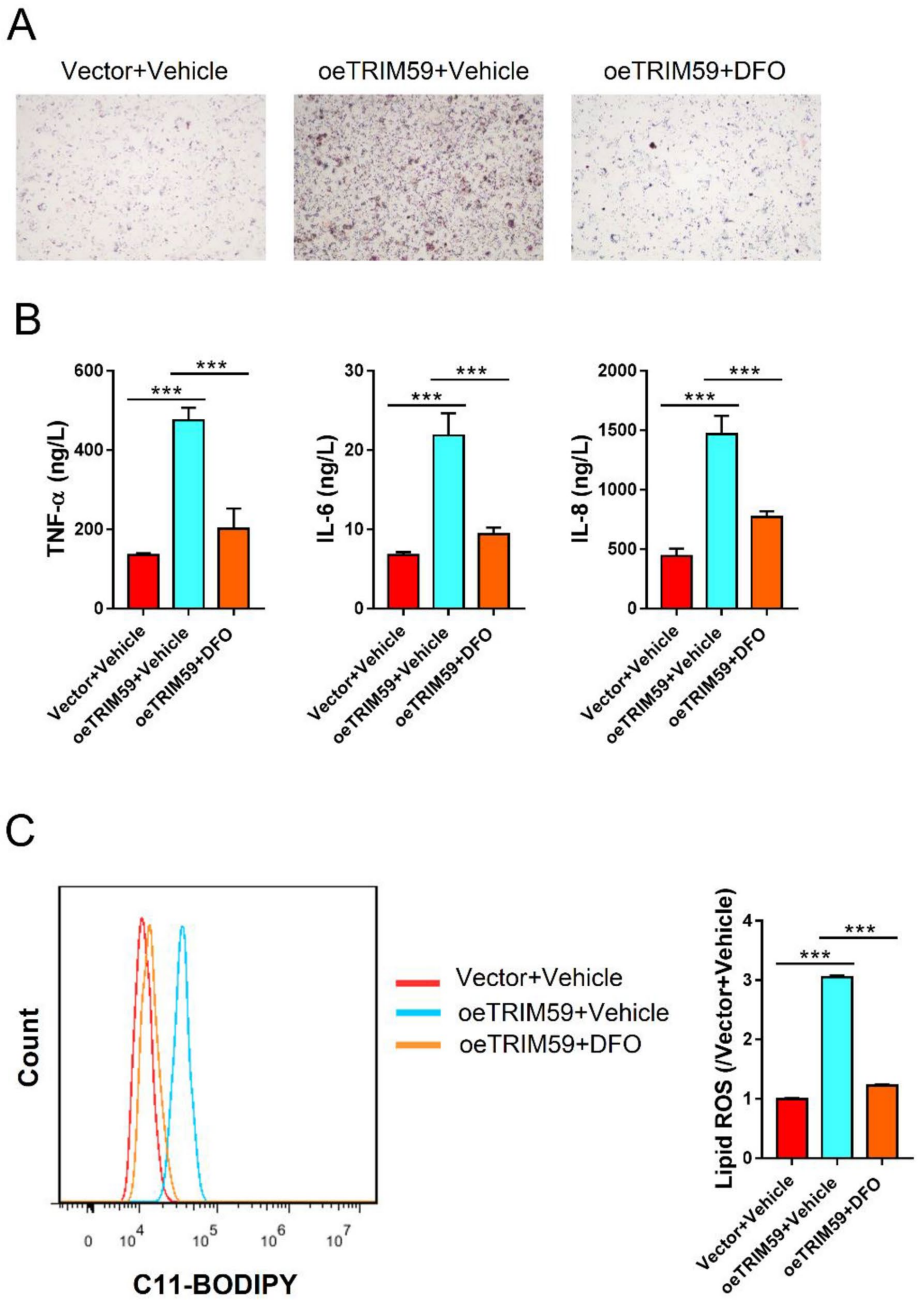
Deferoxamine inhibited TRIM59-induced steatosis and inflammation in L02 cells. L02 cells were treated with oeTRIM59 adenovirus and 100 M ferroptosis inhibitor deferoxamine (DFO) or Vehicle (DMSO) for 24 h. **A** Oil Red O staining detected the level of steatosis in L02 cells (magnification: 200x). **B** ELISA assay detected the level of TNF-α, IL-6, and IL-8. **C** Flow cytometry detected the level of lipid ROS. ***P < 0.001, *n* = 3

Since ferroptosis might contribute to the progression of NAFLD, we adopted a specific inhibitor of ferroptosis, deferoxamine (DFO), to explore the correlation between TRIM59 and ferroptosis. Results indicated that the elevated expression of TRIM59 could promote steatosis in L02 cells, whereas the application of DFO could notably reverse this effect (Fig. 4A). Meanwhile, the level of TNF-α, IL-6 and IL-8 was significantly promoted by oeTRIM59 but decreased by DFO (*P* < 0.05) (Fig. 4B). Flow cytometry revealed that lipid ROS was significantly elevated by TRIM59 and was significantly decreased by DFO (*P* < 0.05) (Fig. 4C). These findings suggested that the inhibition of ferroptosis could reverse TRIM59-induced steatosis and inflammation in NAFLD cell model.

### TRIM59 could promote the ubiquitination of GPX4

**Fig. 5:**
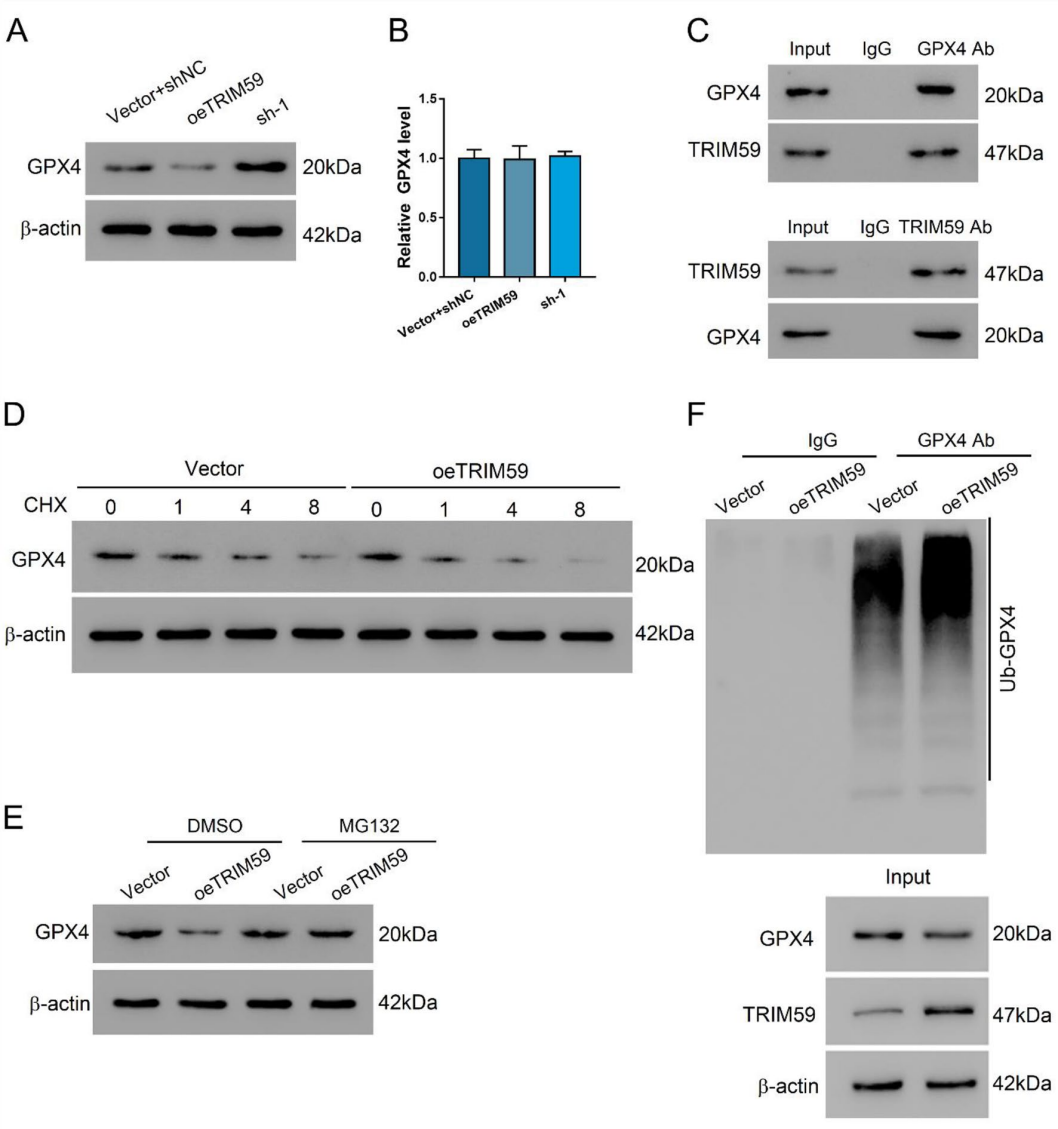
TRIM59 could interact with GPX4 and promote its ubiquitination. **A**, **B** The protein (**A**) and mRNA (**B**) levels of GPX4 in L02 cells transduced with shTRIM59 adenovirus or oeTRIM59 lentivirus. **C** Co-IP assay of the interaction between TRIM59 and GPX4. **D** The expression of GPX4 in L02 cells transduced with oeTRIM59 or vector and treated with 20 mM cycloheximide (CHX). **E** The expression of GPX4 in L02 cells transduced with oeTRIM59 or vector and treated with 10 μM MG132 or DMSO for 20 h. **F** The ubiquitination level of GPX4 in L02 cells transduced with oeTRIM59 or vector

To explore the correlation between TRIM59 and ferroptosis, we investigated the interaction between TRIM59 and GPX4, the key factor of ferroptosis. The overexpression of TRIM59 could suppress the protein level of GPX4 and its inhibition exerted opposite effects (Fig. 5A). Meanwhile, the mRNA level of GPX4 was not affected by the altered expression of TRIM59, indicating that TRIM59 could affect the post-transcriptional modification of GPX4 (*P* > 0.05) (Fig. 5B). Co-IP assay revealed that TRIM59 could interact with GPX (Fig. 5C). Moreover, with the application of cycloheximide (CHX), we found that TRIM59 could enhance the degradation of GPX4 (Fig. 5D). When MG132 was applied to inhibit the activity of proteasome, TRIM59 failed to decrease the protein expression of GPX4 (Fig. 5E). Co-IP assay revealed that the TRIM59 could promote the ubiquitination of GPX4 and thus decrease its expression (Fig. 5F). These results indicated that TRIM59 could interact with GPX4 and promote its ubiquitination.

### Overexpression of GPX4 reversed the effects mediated by TRIM59

**Fig. 6:**
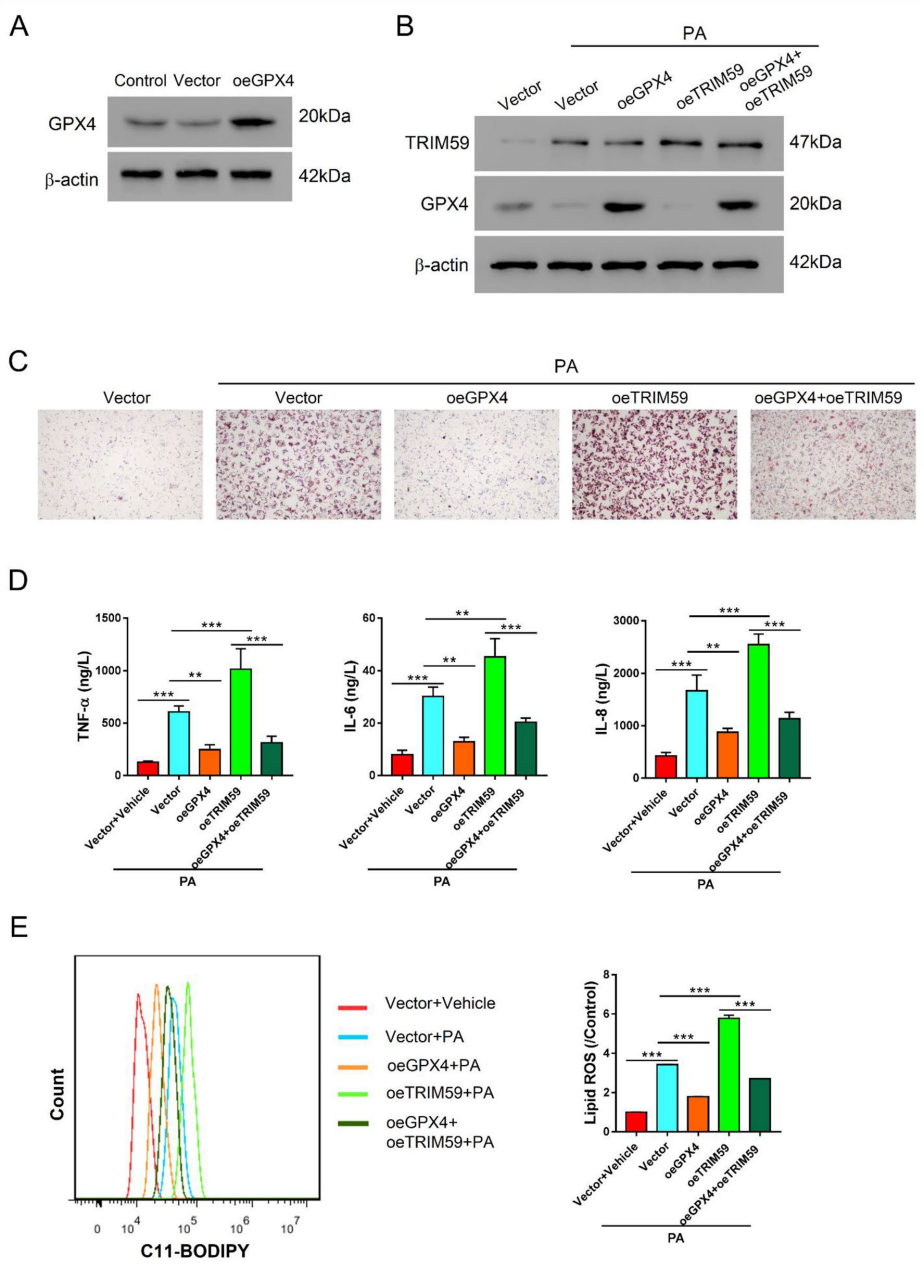
Overexpression of GPX4 reversed the effects mediated by TRIM59. **A** The expression of GPX4 in L02 cells transduced with oeGPX4 lentivirus. **B**–**E** L02 cells were treated with 0.4 mM PA, oeGPX4 lentivirus, and oeTRIM59 lentivirus. **B** Western blot detected the expression of TRIM59 and GPX4. **C** Oil red O staining detected the level of steatosis in L02 cells (magnification: 200×). **D** ELISA assay detected the level of TNF-α, IL-6, and IL-8. **E** Flow cytometry detected the level of lipid ROS. **P < 0.01, ***P < 0.001

To further explore the association between TRIM59 and GPX4 in NAFLD, we transduced overexpressed GPX4 lentivirus (oeGPX4) in L02 cells, which notably promoted the expression of GPX4 (Fig. 6A). With the presence of PA, the transduction of oeGPX4 increased the expression of GPX4 but did not affect the expression of TRIM59 (Fig. 6B). Besides, oeGPX4 could reverse the depletion of GPX4 that was mediated by the overexpression of TRIM59 (Fig. 6B). In L02 cells, the elevation of GPX4 markedly ameliorates the steatosis in the presence of oeTRIM59 or not (Fig. 6C). Moreover, oeGPX4 could significantly decrease the level of TNF-α, IL-6 and IL-8, which could be promoted by the overexpression of TRIM59 (*P* < 0.05) (Fig. 6D). Additionally, the level of lipid ROS was significantly reduced by overexpressed GPX4, which reversed the effects mediated by TRIM59 (*P* < 0.05) (Fig. 6E). These findings suggested that the overexpression of GPX4 exerted opposite effects of TRIM59. GPX4 might serve as the downstream of TRIM59 in NAFLD.

### Knockdown of TRIM59 attenuated HFD-induced steatosis and ferroptosis in NAFLD mice

**Fig. 7:**
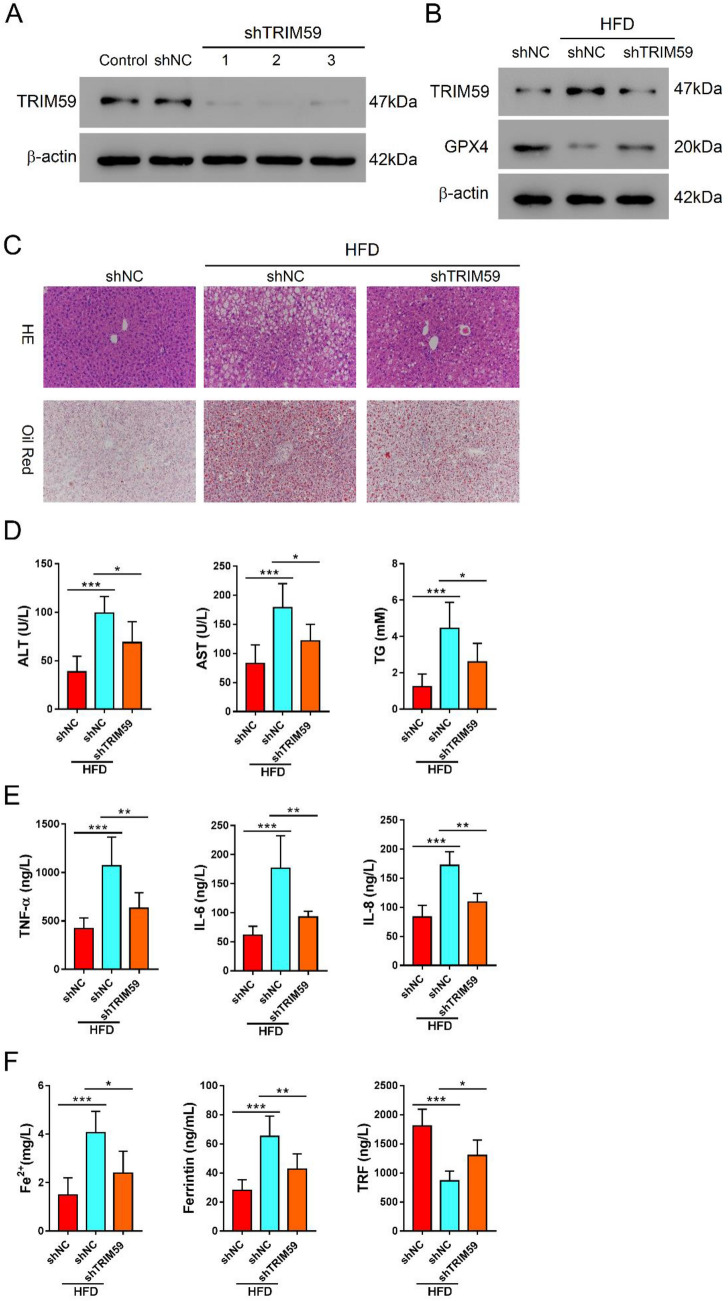
Knockdown of TRIM59 attenuated HFD-induced steatosis and ferroptosis in NAFLD mice. **A** The expression of TRIM59 in AML12 cells transduced with shTRIM59 adenovirus. **B**–**F** NAFLD mice model was constructed with the application of high-fatty diet. Mice were intravenously treated with shTRIM59 and control adenovirus for 8 weeks. **B** Western blot detected the expression of TRIM59 and GPX4 in liver tissues of mice. **C** HE and oil red O staining of liver tissues. **D** The level of AST, ALT, and TG in NAFLD mice. **E** ELISA detected the level of TNF-α, IL-6, IL-8 in NAFLD mice. **F** The level of Fe^2+^, ferritin, and TRF in NAFLD mice. *P < 0.05, **P < 0.01, ***P < 0.001

Further, we verified the role of TRIM59 in NAFLD mice model. The shTRIM59 adenovirus was transduced in mouse AML12 cells and markedly inhibited the expression of TRIM59 (Fig. 7A). Mice were treated with high-fatty diet (HFD) to induce NAFLD and applied with shTRIM59 adenovirus. Western blot showed that the expression of TRIM59 was increased, whereas that of GPX4 was decreased by HFD in liver tissue of mice; the knockdown of TRIM59 could reduce the expression of TRIM59 and elevate the expression of GPX4 (Fig. 7B). Meanwhile, the level of steatosis was promoted by HFD but decreased by the inhibition of TRIM59 (Fig. 7C). In NAFLD mice, the level of ALT, AST, and TG was significantly increased by HFD and decreased by the knockdown of TRIM59 (*P* < 0.05) (Fig. 7D). Besides, HFD significantly promoted the level of inflammatory cytokines including TNF-α, IL-6 and IL-8, which could be suppressed by the knockdown of TRIM59 (*P* < 0.05) (Fig. 7E). Moreover, the level of Fe^2+^ and ferritin was significantly promoted by HFD and decreased by shTRIM59 (*P* < 0.05) (Fig. 7F). In contrast, the level of TRF was significantly reduced by HFD but promoted by shTRIM59 (*P* < 0.05) (Fig. 7F). These results indicated that the inhibition of TRIM59 could ameliorate the steatosis and suppress ferroptosis in NAFLD mice model.

## Discussion

NAFLD has become the most common liver disease around the world [34]. In this study, we found that TRIM59 was highly expressed in NAFLD tissues compared with normal liver tissues. The inhibition of TRIM59 could inhibit the steatosis and inflammation in NAFLD cell model, whereas its overexpression exhibited reversed effects. The application of ferroptosis inhibitor, DFO, could markedly ameliorate steatosis and inflammation, which was mediated by overexpressed TRIM59. Besides, TRIM59 was demonstrated to interact with GPX4 and promoted its ubiquitination. The overexpression of GPX4 could significantly reverse the pathogenic effects of TRIM59 in NAFLD. Additionally, the inhibition of TRIM59 appeared to be a promising strategy to ameliorate NAFLD in mice model. Our study revealed a novel molecular mechanism underlying the pathogenesis of NAFLD and provided a potential target for NAFLD treatment.

TRIM protein family consists of more than 70 members in humans and is a subfamily of the RING-type E3 ubiquitin ligase family [16]. Several members of TRIM family are strong regulators of cellular activity and are involved in ubiquitination of other proteins. TRIM proteins can regulate other proteins like receptors, enzymes, intracellular signal transducers and transcription factors that play important role in innate immunity [13, 16]. Here, we detected the expression of several TRIM family members in NAFLD tissues and normal tissues, in which TRIM59 expression was significantly associated with the severity of NAFLD. Further experiments verified that TRIM59 was highly expressed in severe NAFLD tissues. Therefore, TRIM59 might play a pathogenic role in the development of NAFLD.

Steatosis is implicated to be the initiation of NAFLD disease, in which more than 5% of hepatocytic steatosis is defined as NAFLD [35]. During the early stage of NAFLD, the liver function is usually unaffected. However, when NAFLD progresses to NASH, whose hallmarks are liver inflammation and fibrosis, the liver function is impaired and the risk of cirrhosis and hepatocellular carcinoma is dramatically increased [36, 37]. The reduction of hepatic steatosis and inflammation by vitamin E, Mediterranean diet, and herbal compounds is proven to benefit NAFLD patients [38–40]. However, no specific medicine has been approved for the treatment of NAFLD. In our study, we found that TRIM59 could promote the steatosis and inflammation in NAFLD cell model, and the inhibition of TRIM59 was a promising strategy to ameliorate NAFLD both in vitro and in vivo. Therefore, our study provided a novel target for the treatment of NAFLD.

A previous study indicated that ferroptosis played a critical role for the development NAFLD [41]. The application of ferrostatin-1, a ferroptosis inhibitor, significantly attenuated the ferroptosis and NASH in L02 cells [42]. Besides, the inhibition of ferroptosis was demonstrated to protect hepatocytes from necroptosis and suppress the subsequent inflammation [43]. Dehydroabietic acid could improve NAFLD via inhibiting ferroptosis in mice model [44]. Our study revealed that the level of Fe^2+^ and ferritin was significantly promoted, whereas that of TRF was significantly reduced in NAFLD tissues. The application of DFO, the ferroptosis inhibitor, significantly attenuated TRIM59-induced steatosis and inflammation in NAFLD. Therefore, the inhibition of ferroptosis is a promising strategy for NAFLD therapy.

GPX4 is the key enzyme that protects cells against lipid peroxidation. The suppression of GPX4 could lead to the ferroptosis [45]. A previous study indicated that the increased level of GPX4 was associated with the reduction of NAFLD severity [46]. Moreover, the increased GPX4 could attenuate lipid accumulation [47]. Herein, our study revealed that TRIM59 shared the similar expression pattern with Fe^2+^ and ferritin, indicating the intimate association between TRIM59 and ferroptosis. Previous studies have suggested that TRIM59 exerted functions via its E3 ligase activity [48]. Our further experiments showed that TRIM59 could interact with GPX4 and reduced its expression through promoting its ubiquitination. The overexpression of GPX4 could reverse TRIM59-induced steatosis, ferroptosis and inflammation in vitro. GPX4 expression was decreased in liver tissues of mice fed with HFD; the knockdown of TRIM59 could elevate GPX4 expression and decrease steatosis.

Therefore, our study revealed a novel TRIM59/GPX4 signaling pathway in the pathogenesis of NAFLD.

## Conclusion

To sum up, our study revealed that TRIM59 could promote steatosis and ferroptosis in NAFLD via enhancing GPX4 ubiquitination. TRIM59 could be a potential target for NAFLD treatment.

## Supplementary Information

Below is the link to the electronic supplementary material.

Supplementary file1 (DOCX 170 KB)

Supplementary file2 (PDF 1504 KB)
