# Supplementary material for: TRIM59 promotes steatosis and ferroptosis in non-alcoholic fatty liver disease via enhancing GPX4 ubiquitination
Source: Hum Cell. 2022 Nov 22;36(1):209–22. doi: 10.1007/s13577-022-00820-3 (PMC9813033; doi:10.1007/s13577-022-00820-3)
Supplement: Supplementary file 1 — Supplementary file1 (DOCX 170 KB) [file 13577_2022_820_MOESM1_ESM.docx]

**
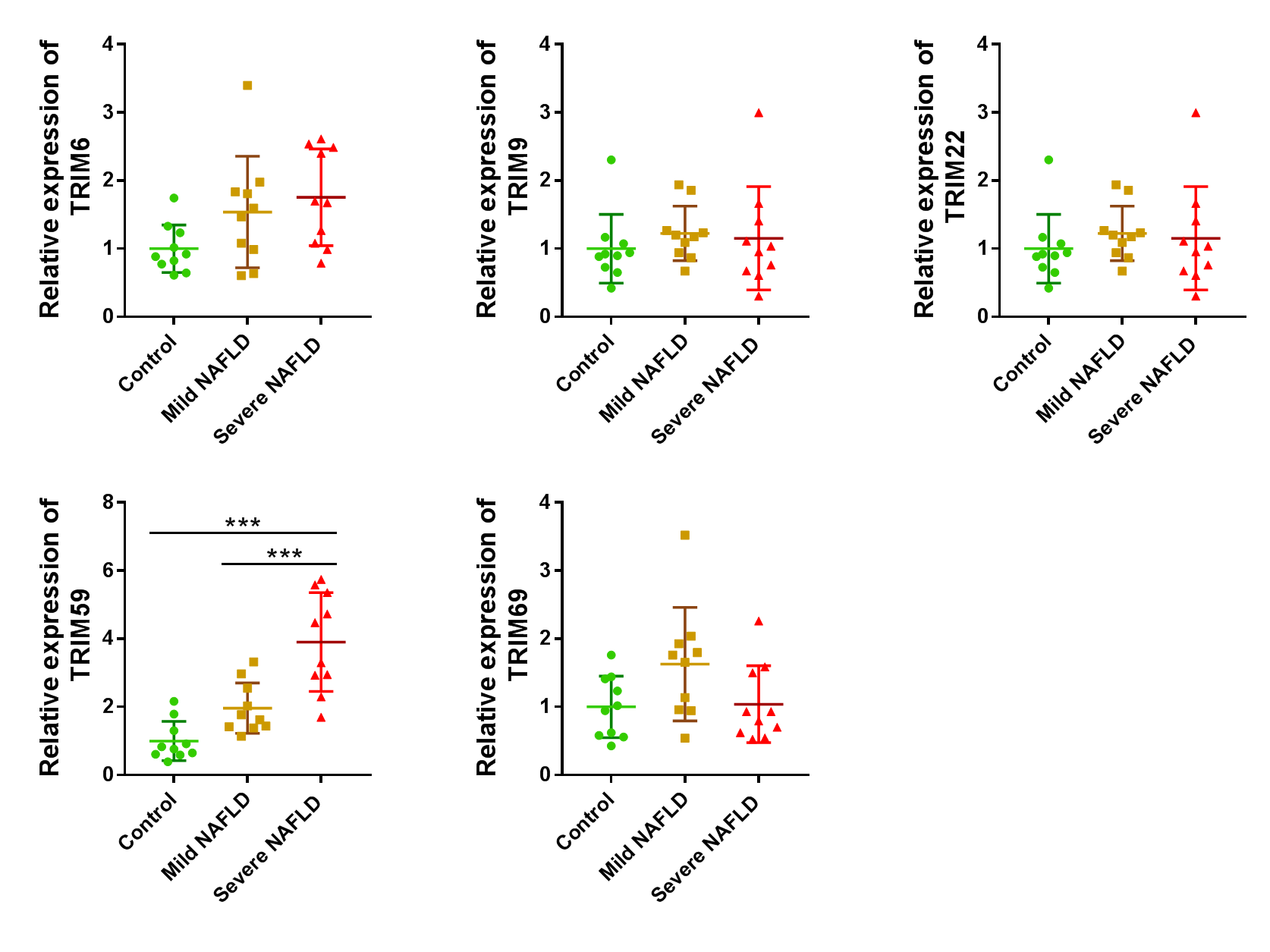
**

**Figure S**1. The mRNA level of several TRIM family members in 10 cases of normal, mild and severe fatty liver tissues. ***P<0.001.

**Table S1.** Primer sequences for real-time PCR.

| **Gene** | **Forward primer** | **Reverse primer** |
| --- | --- | --- |
| TRIM59 | 5' TTGTCACCTGCCCTGAAC 3' | 5' TCCTTATCGCCTTGGATC 3' |
| TRIM6 | 5' GGTCATTTGCTGGCTTTG3' | 5' GCTTCCTGCTCCTCGTTC 3' |
| TRIM9 | 5' GCAAACACTCCAGCCACG 3' | 5' TCTGATCTCGAACCACCTTC 3' |
| TRIM22 | 5' CAAACATTCCGCATAAAC 3' | 5' ATCCAGCACATTCACCTCAC 3' |
| TRIM69 | 5' GCCTTGAATGAGGAGATG 3' | 5' AGGGTCCAGAGTTAGTGG 3' |
| GPX4 | 5' GCAAGACCGAAGTAAACTACAC 3' | 5' CTCCTGCTTCCCGAACTG 3' |
| β-actin | 5' TGGCATCCACGAAACTAC 3' | 5' CTTGATCTTCATGGTGCTG 3' |

**Table S2.** Antibody list.

| **Primary antibody** | **Company** | **Catalog No.** |
| --- | --- | --- |
| TRIM59 | Abcam | ab125066 |
| GPX4 | Abcam | ab125066 |
| Ub | Abcam | ab7780 |
| GAPDH | Cell Signaling Technology | #5174 |

**Table S3.** Target sequences of human TRIM59 shRNAs.

| **shRNA** | **Target sequence** |
| --- | --- |
| sh-1 | 5’ GGAAGCTGTTCTCCAGTAT 3’ |
| sh-2 | 5’ GAAGAGTCTCCACTTAAAT 3’ |
| sh-3 | 5’ GAATGGAGCAGAACAGAAA 3’ |

**Table S4.** Target sequences of mouse TRIM59 shRNAs.

| **shRNA** | **Target sequence** |
| --- | --- |
| shTRIM59-1 | 5’ GCTTCTACTGGCATAGAAT 3’ |
| shTRIM59-2 | 5’ GGAGATAAGGGAACAACAA 3’ |
| shTRIM59-3 | 5’ CCAGCCTGTTGAAATTTAT 3’ |
