# Supplementary figures and images for: TRIM59 promotes steatosis and ferroptosis in non-alcoholic fatty liver disease via enhancing GPX4 ubiquitination
Source: Hum Cell. 2022 Nov 22;36(1):209–22. doi: 10.1007/s13577-022-00820-3 (PMC9813033; doi:10.1007/s13577-022-00820-3)

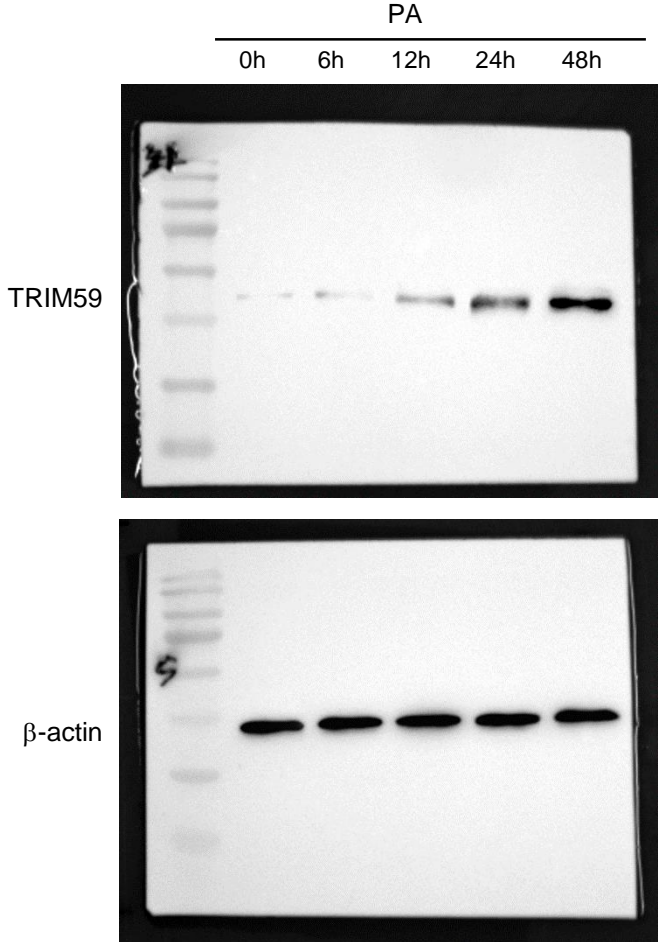

Fig. 2A

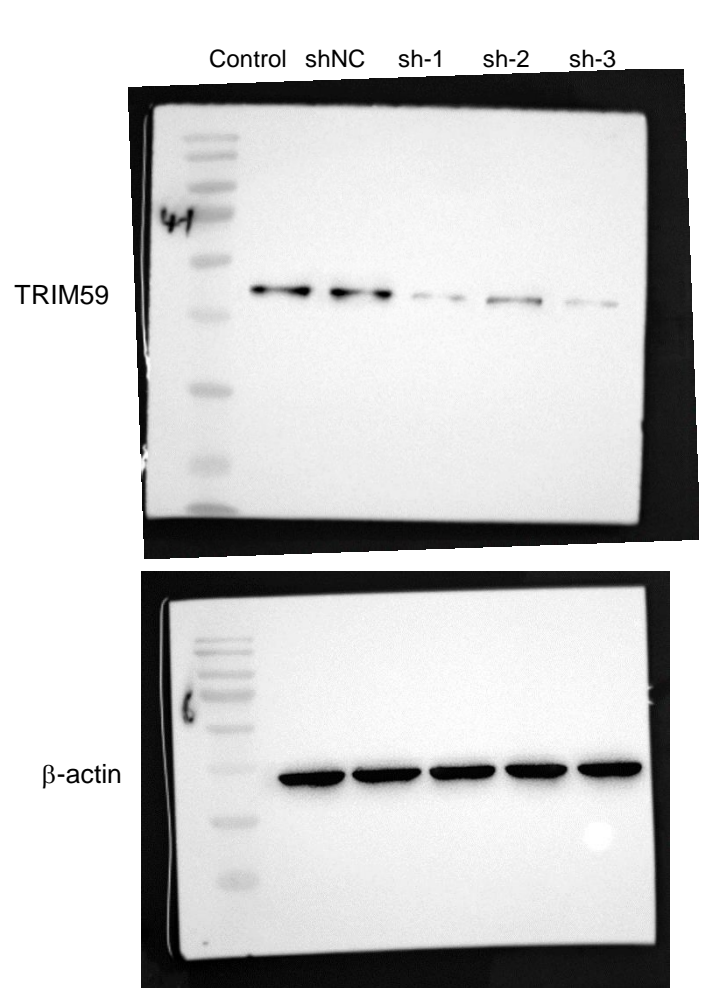

Fig. 2B

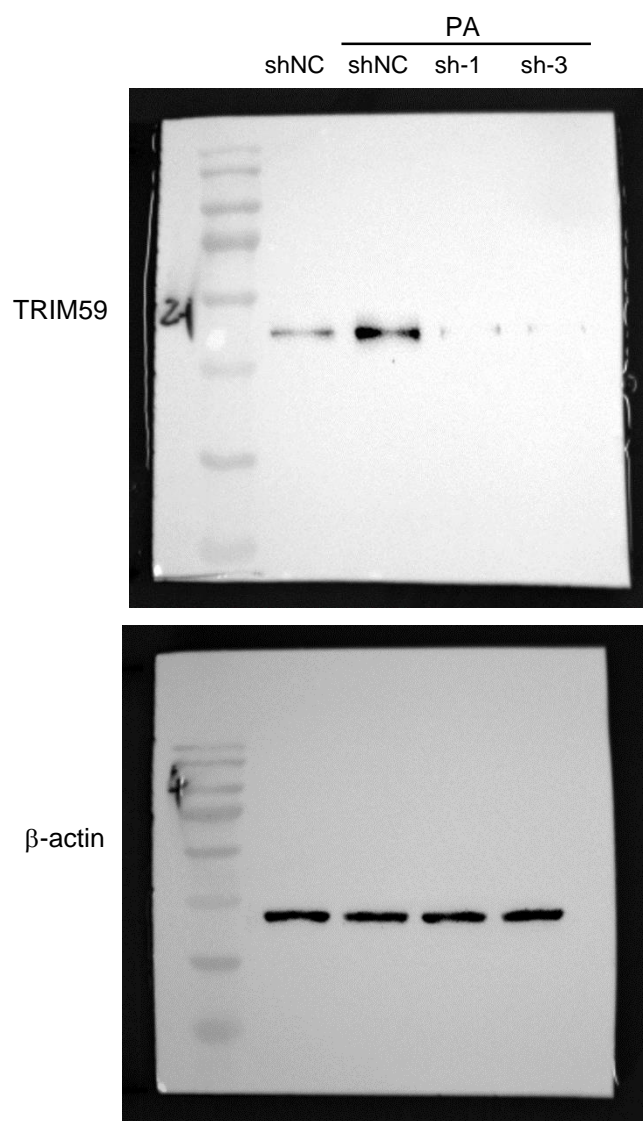

Fig. 2C

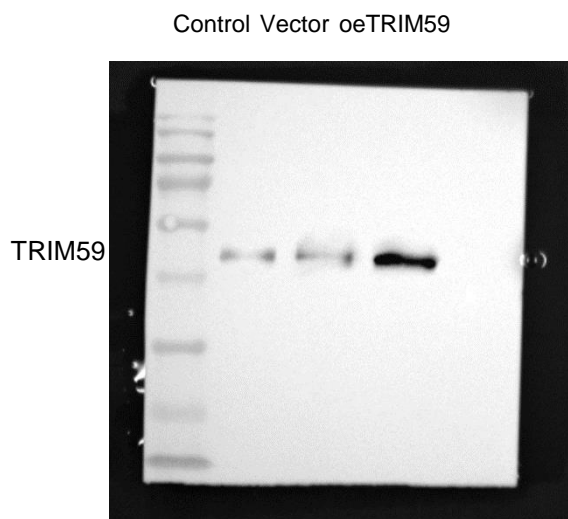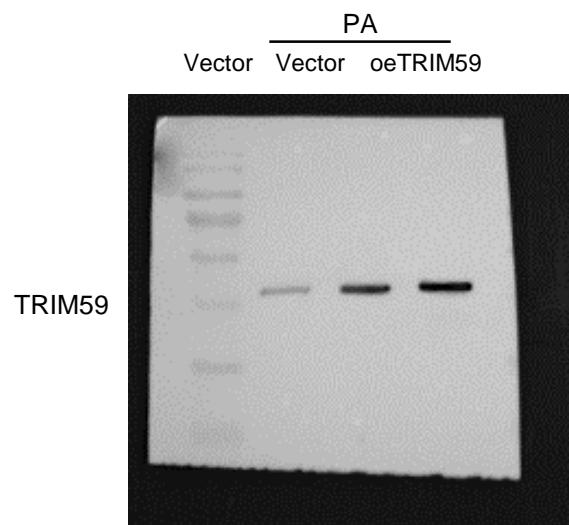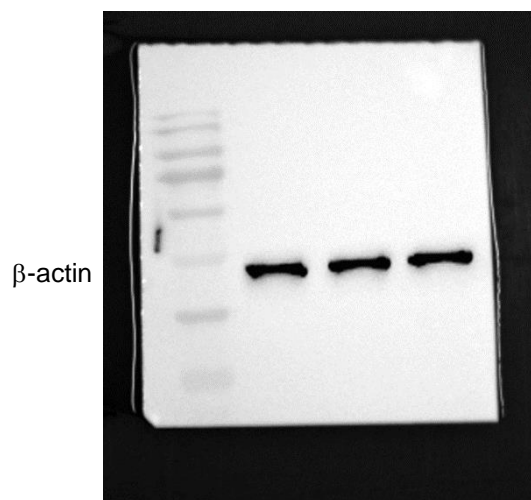

Fig. 3A

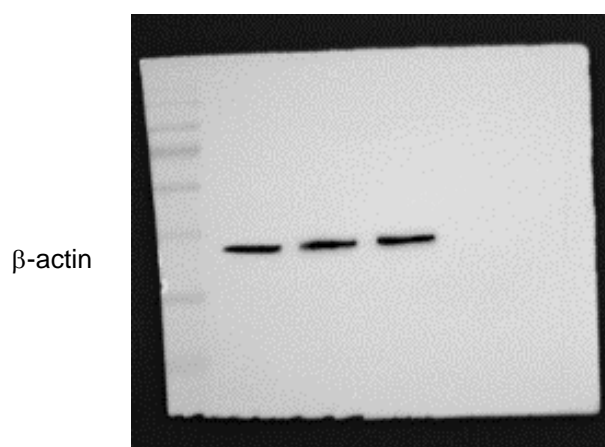

Fig. 3B

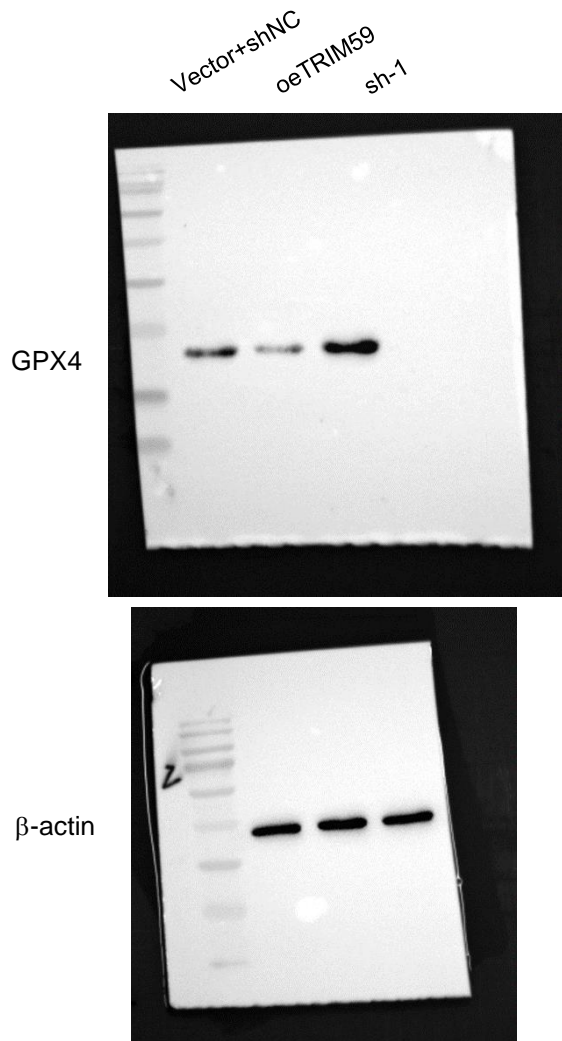

Fig. 5A

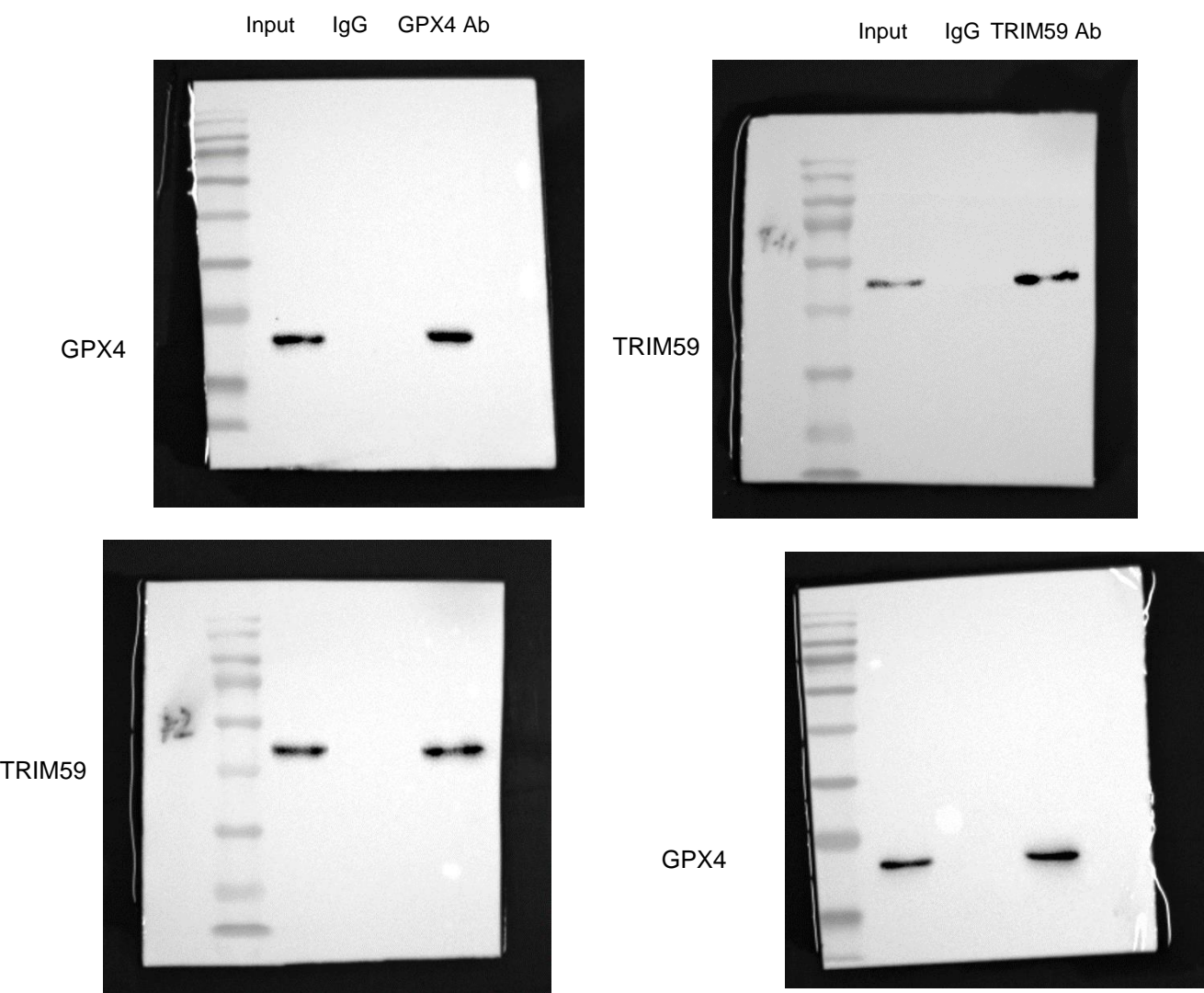

Fig. 5C

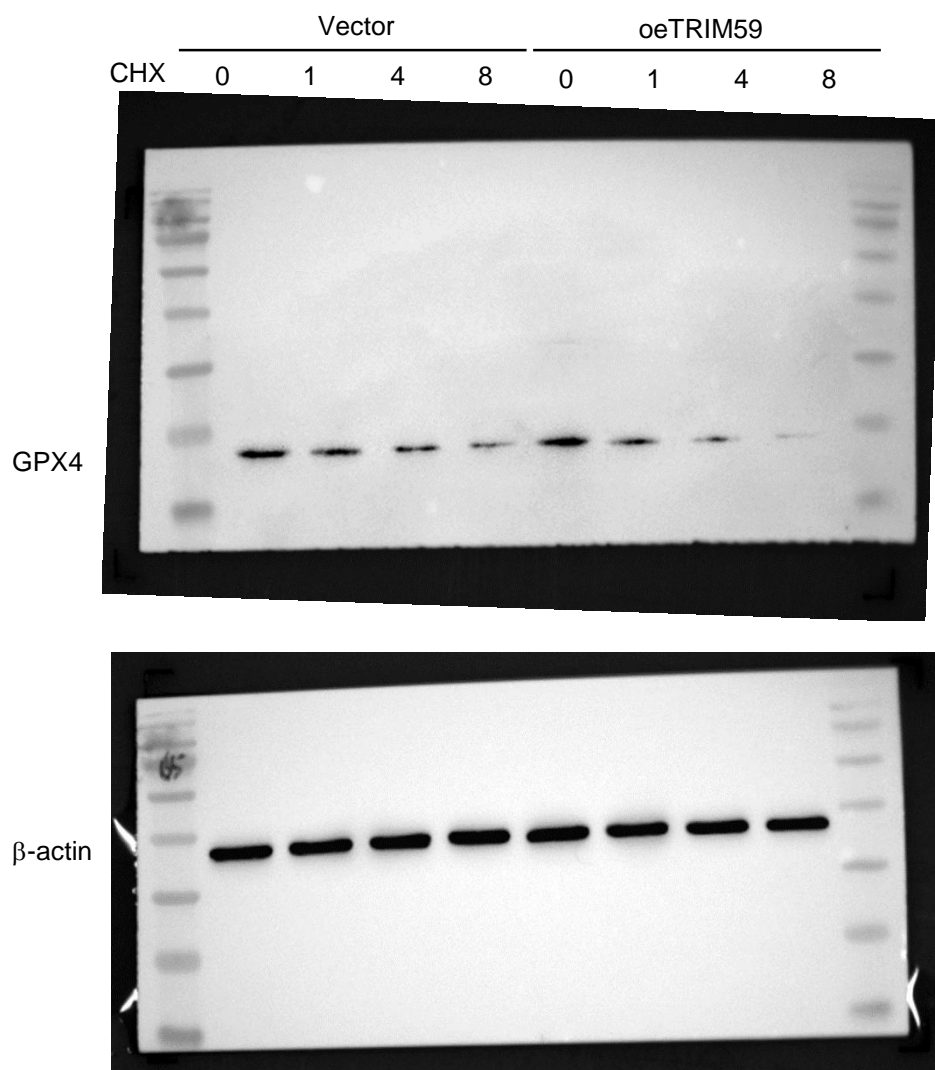

Fig. 5D

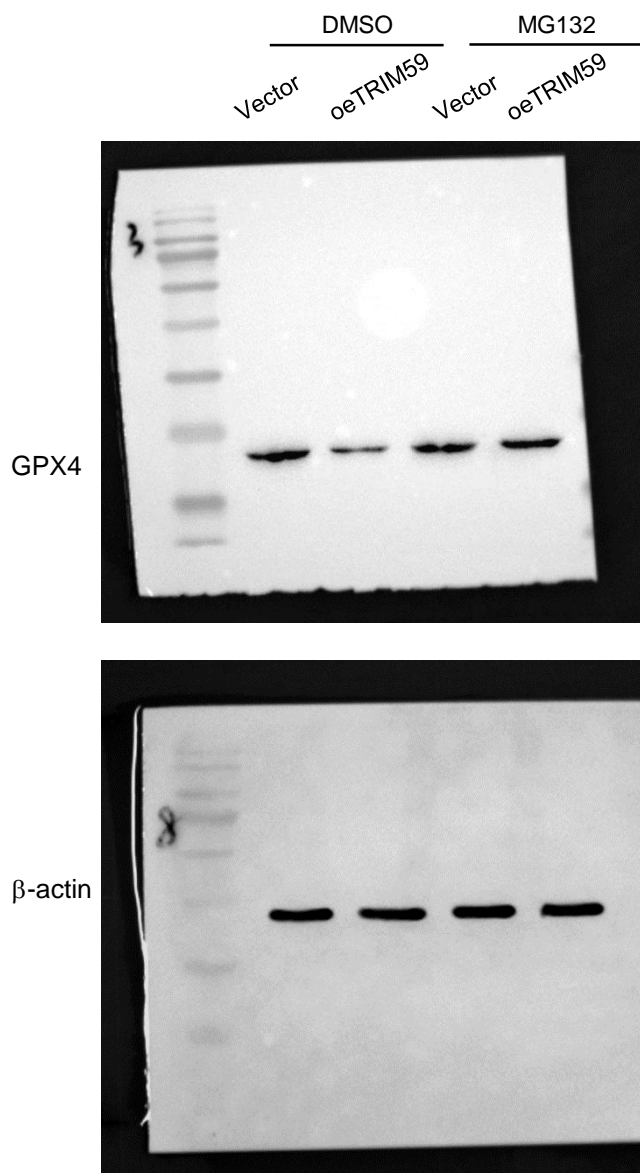

Fig. 5E

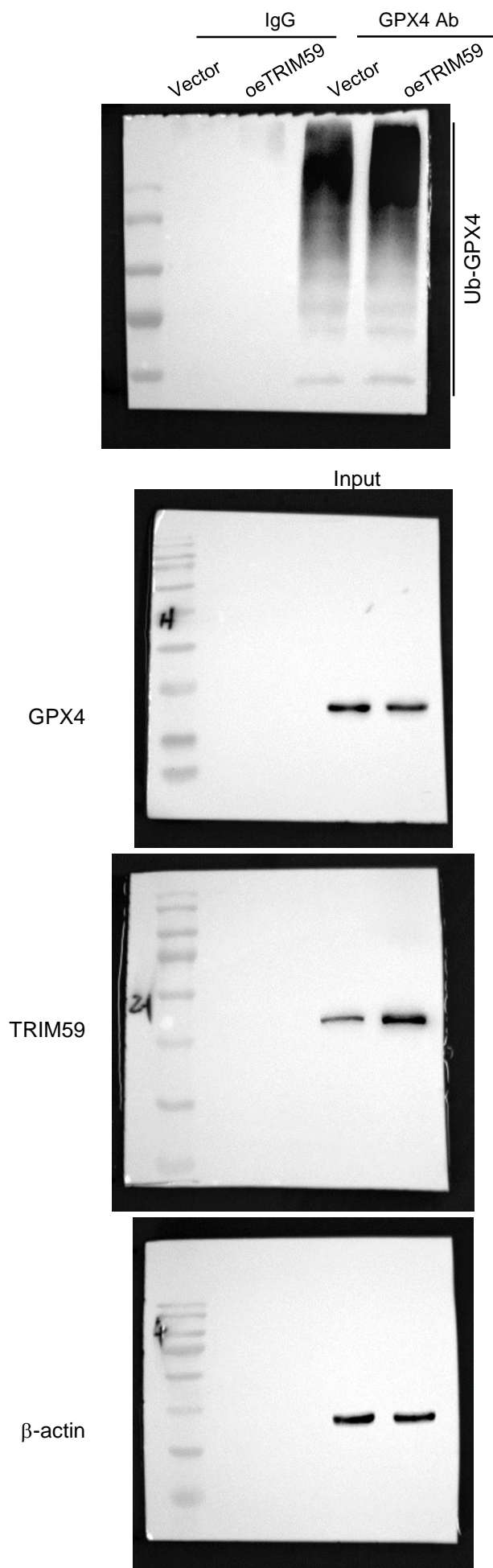

Fig. 5F

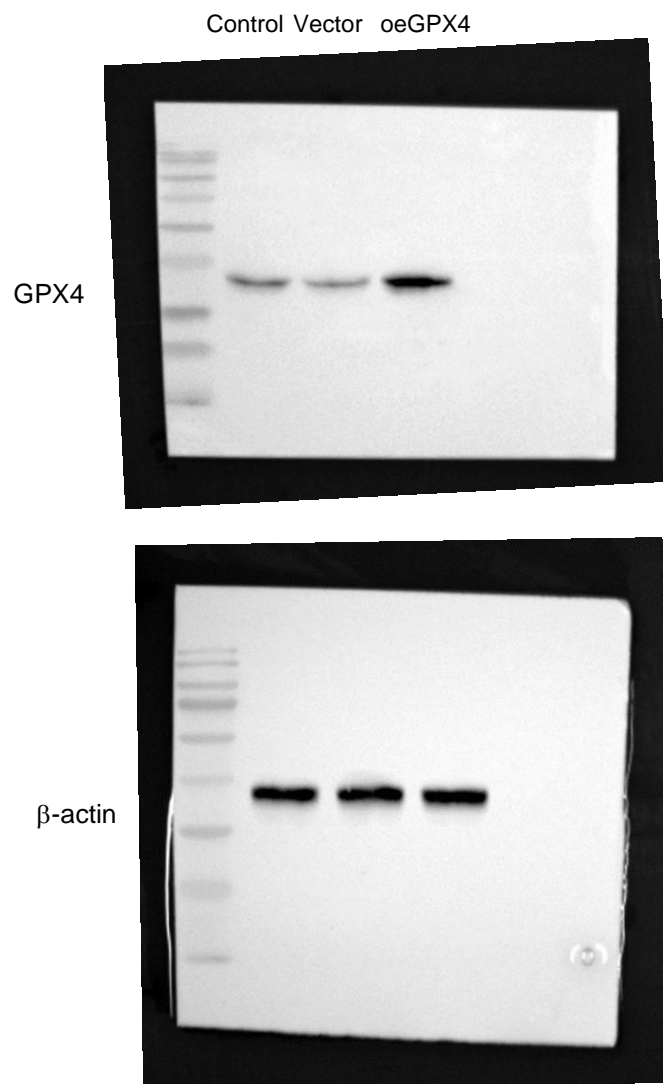

Fig. 6A

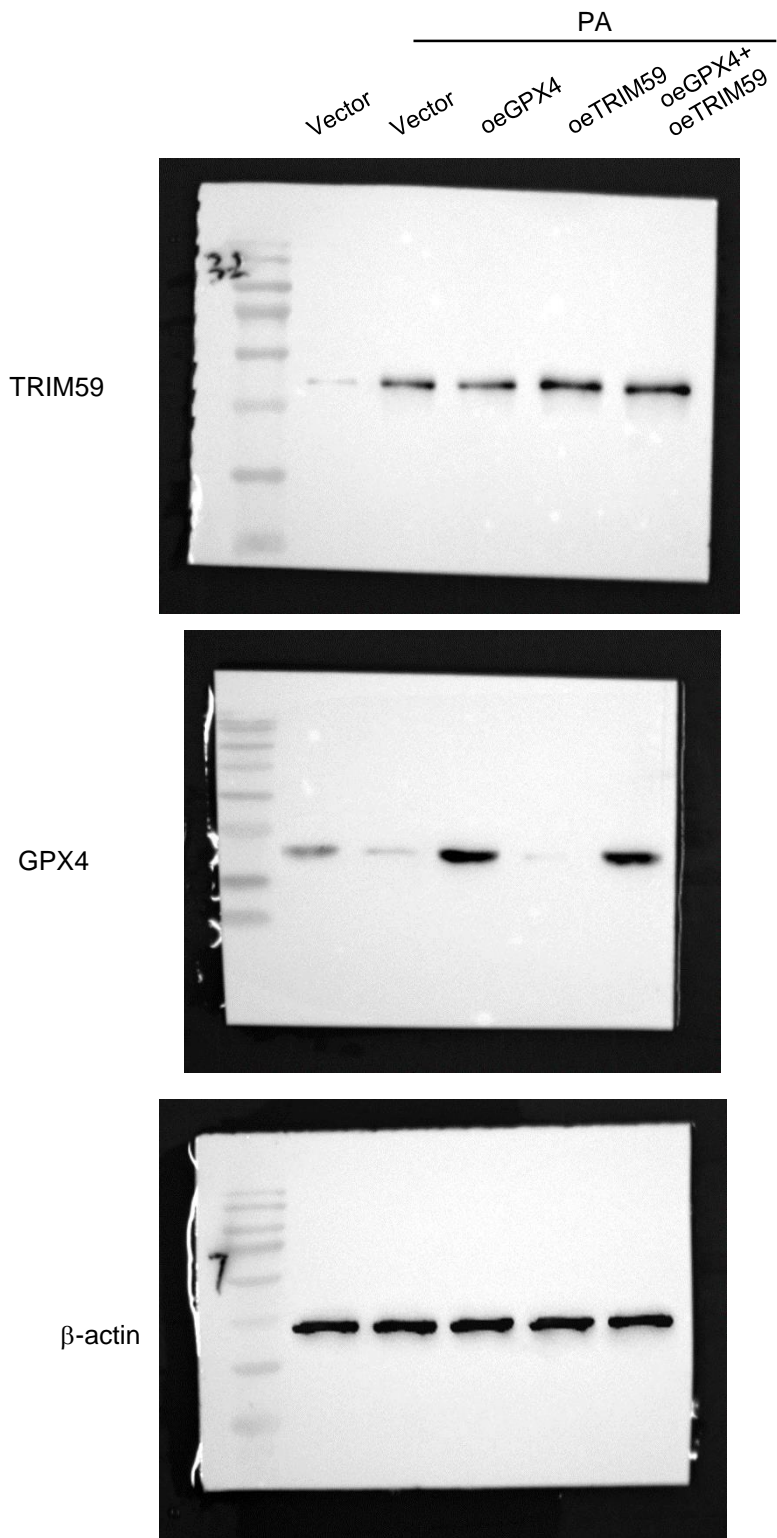

Fig. 6B

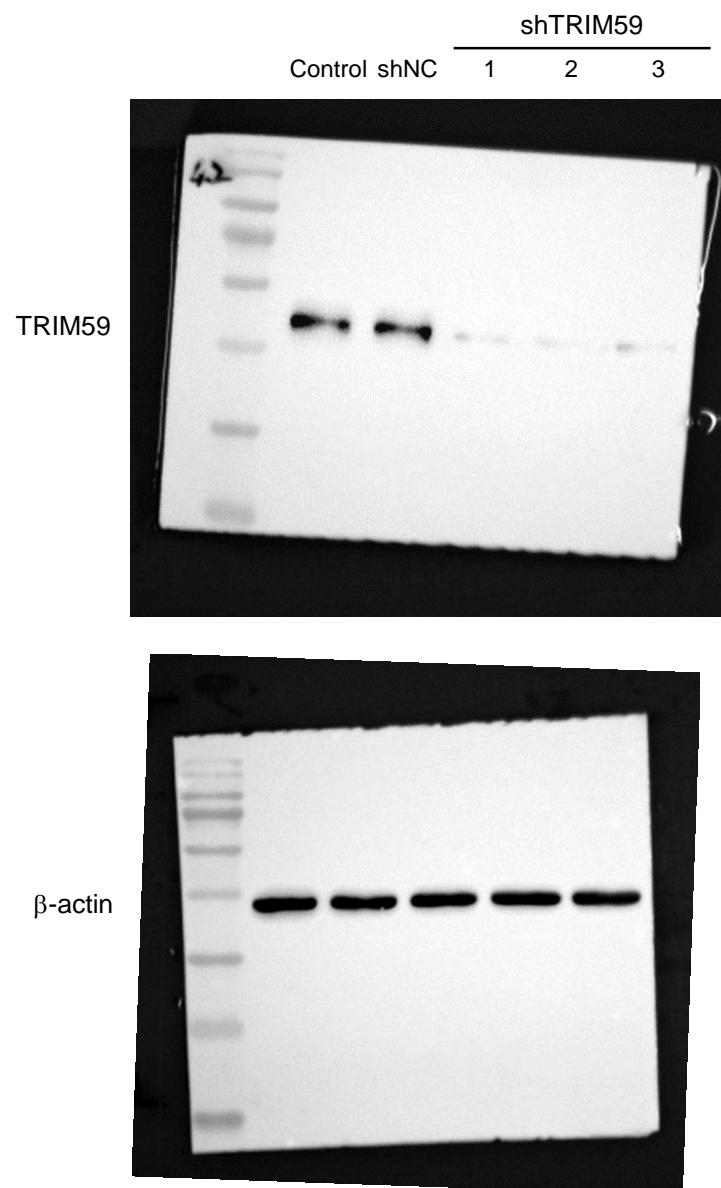

Fig. 7A

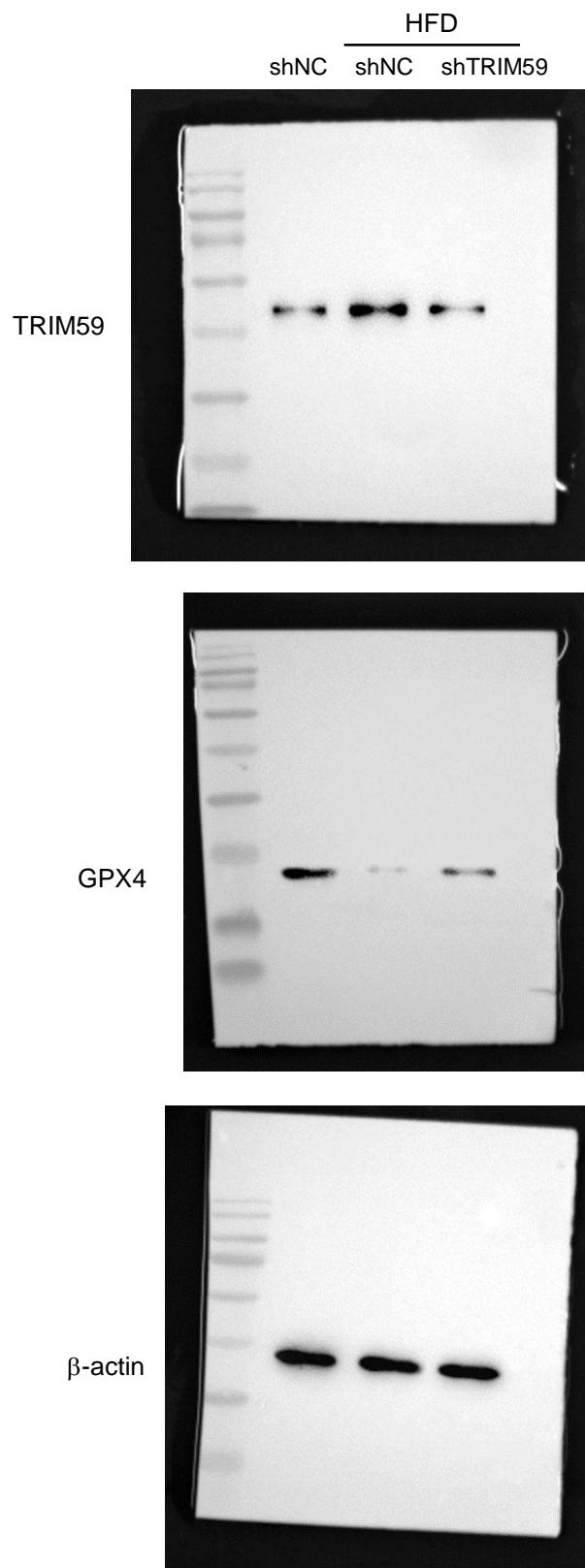

Fig. 7B

Supplement: Supplementary file 2 — Supplementary file2 (PDF 1504 KB) [file 13577_2022_820_MOESM2_ESM.pdf]
